# Decreased sensitivity to paroxetine-induced inhibition of peripheral blood mononuclear cell growth in depressed and antidepressant treatment-resistant patients

**DOI:** 10.1038/tp.2016.90

**Published:** 2016-05-31

**Authors:** S Rzezniczek, M Obuchowicz, W Datka, M Siwek, D Dudek, K Kmiotek, K Oved, N Shomron, D Gurwitz, A Pilc

**Affiliations:** 1 Department of Neurobiology, Institute of Pharmacology Polish Academy of Science, Krakow, Poland; 2 Department of Affective Disorders, Chair of Psychiatry, Jagiellonian University Medical College, Krakow, Poland; 3 Department of Human Molecular Genetics & Biochemistry, Sackler Faculty of Medicine, Tel Aviv University, Tel Aviv, Israel; 4 Department of Cell and Developmental Biology, Tel Aviv University, Sackler Faculty of Medicine, Tel Aviv, Israel; 5 Sagol School of Neuroscience, Tel Aviv University, Tel Aviv, Israel; 6 Institute of Public Health, Faculty of Health Sciences, Jagiellonian University, Krakow, Poland

## Abstract

Major depression disorder (MDD) is the most widespread mental disorder. Selective serotonin reuptake inhibitors (SSRIs) are used as first-line MDD treatment but are effective in <70% of patients. Thus, biomarkers for the early identification of treatment-resistant (TR) MDD patients are needed for prioritizing them for alternative therapeutics. SSRI-induced inhibition of the growth of peripheral blood mononuclear cells (PBMCs) is mediated via their target, the serotonin transporter (SERT). Here, we examined whether antidepressant drug-induced inhibition of the growth of PBMCs differed between MDD patients and healthy controls. PBMCs from well-characterized 33 treatment-sensitive (TS) and 33 TR MDD patients, and 24 healthy volunteers were studied. Dose-dependent inhibition of PBMCs growth was observed for both the non-SSRI antidepressant mirtazapine and the SSRI antidepressant paroxetine. Significantly lower sensitivities to 20 μm paroxetine were observed in MDD compared with control PBMCs prior to treatment onset (13% and 46%, respectively; *P<*0.05). Following antidepressant drug treatment for 4 or 7 weeks, the *ex vivo* paroxetine sensitivity increased to control levels in PBMCs from TS but not from TR MDD patients. This suggests that the low *ex vivo* paroxetine sensitivity phenotype reflects a state marker of depression. A significantly lower expression of *integrin beta-3* (*ITGB3*), a co-factor of the SERT, was observed in the PBMCs of MDD patients prior to treatment onset compared with healthy controls, and may explain their lower paroxetine sensitivity. Further studies with larger cohorts are required for clarifying the potential of reduced PBMCs paroxetine sensitivity and lower *ITGB3* expression as MDD biomarkers.

## Introduction

Major depressive disorder (MDD) is among the most common chronic human diseases with an estimated 350 million people of all ages affected globally.^1^ Selective serotonin reuptake inhibitors (SSRIs) are most commonly employed as a first-line treatment for MDD. However, treatment resistance occurs in >30% of MDD patients and this outcome requires alternative antidepressants.^2, 3^ Typically, 2–4 weeks are required to evaluate the response to antidepressants, and the clinical guidelines accordingly recommend waiting for at least 4–8 weeks before switching to another antidepressant drug when favorable response is not achieved. This long waiting period, combined with the high rate of SSRI non-response, increase adverse effect and suicide risks and contribute to the high societal cost of MDD.^4^ Therefore, an unmet need to identify treatment resistance biomarkers, preferably in the blood (blood cells, plasma or serum), which will enable the early identification of MDD patients who are likely to be resistant to treatment with SSRI antidepressants exists. Such a biomarker would also allow for the prioritization of these patients for treatment with alternative antidepressant drugs and more intensive clinical follow-up.

Circulating human lymphocytes express functional serotonin transporter (SERT; encoded by *SLC6A4*)^5, 6, 7, 8^ that exhibits a pharmacology similar to those of the brain and platelet SERTs^9, 10^ and may therefore serve as an important means to understand the mode of action of antidepressant drugs and treatment resistance in MDD. Human lymphocyte SERT is significantly reduced in MDD patients compared with healthy controls.^6, 7,11^ Furthermore, the SSRI drug fluoxetine significantly increases the number of lymphocytes that express SERT.^12^ In addition, the non-SSRI antidepressant drug mirtazapine exhibits a similar effect on SERT expression, indicating that a complex mechanism that is not directly related to SERT inhibition but may possibly be related to lymphocyte growth inhibition may be functioning.^7, 13^

Recently, Morag *et al.*^14^ reported preliminary observations regarding candidate SSRI response biomarker genes which were detected with genome-wide expression profiling in human lymphoblastoid cell lines (LCLs) from unrelated healthy donors with relatively high or low sensitivities to growth inhibition by the SSRI drug paroxetine. Epstein-Barr virus-immortalized human LCLs are generated from peripheral blood B lymphocytes, retain most of the phenotypic properties of B lymphocytes and exhibit heterogeneity that is specific to the individuals from whom the cells originated.^14, 15^ Thus, human LCLs are suitable for molecular and functional studies and for searching for drug response biomarkers.^14, 15, 16, 17, 18^ SSRI antidepressants, such as paroxetine, fluoxetine, fluvoxamine and citalopram, as well as the tricyclic antidepressant drugs amitriptyline and imipramine have been demonstrated to dose-dependently inhibit the growth of LCLs.^15^ Using a whole-genome expression microarray assay, a 36-fold greater expression of *Close Homolog of LCAM-1* (*CHL1*) was observed in LCLs that exhibit lower SSRI sensitivity *in vitro.*^14^ The *CHL1* gene is located at 3p26.3 and encodes a cell adhesion molecule that is classified as an L-CAM family member. Recent studies have suggested a key role of CHL1 in integrin-mediated embryonic neuronal cell migration.^19, 20, 21^ *CHL1* is specifically expressed in a subpopulation of central and peripheral neurons and glia.^22^ In a subsequent work, Oved *et al.*^17^ demonstrated that chronic treatment of LCLs with paroxetine (1 μm, 21 d) increases the expression of *Integrin beta-3* (*ITGB3*; also known as platelet glycoprotein IIIa and CD61), which is a cell adhesion protein that is required for SERT activity.^23, 24^ The same treatment did not affect *CHL1* expression, and the authors have suggested that the cell adhesion proteins CHL1 and ITGB3 interact in the cell membrane.^17^ It was postulated that the expression levels of both *CHL1* and *ITGB3* may serve as potential SSRI antidepressant response biomarkers.^17^ Indeed, a recent study employing three independent MDD cohorts has reported that certain *CHL1* and *ITGB3* alleles may predict treatment resistance in MDD patients.^25^

The aim of the current study was to explore whether mirtazapine- and paroxetine-mediated growth inhibition of peripheral blood mononuclear cells (PBMCs), which include lymphocytes, monocytes and macrophages, differ between healthy volunteers and MDD patients and between treatment-resistant (TR) and treatment-sensitive (TS) patients. The study also explored whether the candidate biomarker genes *CHL1* and *ITGB3* are differentially expressed in PBMCs from TS and TR MDD patients at the beginning of the study.

## Materials and methods

### Study participants

Blood samples were obtained from 66 clinically well-characterized MDD patients, including 33 TS patients and 33 TR patients, as well as 24 age-matched healthy volunteers. Blood samples from the TS patients were collected three times: at the beginning of the study (TS I); after 4 weeks of treatment (TS II); and after 7 weeks of treatment (TS III). The Hamilton and Montgomery–Asberg Depression Rating Scale (MADRS) scores were determined at the same time points.

### Patient recruitment

Patients (55 women (83.3%) and 11 men (16.7%) above 18 years of age (mean age, 46.7±11.3 years)) who were admitted to the Department of Psychiatry at Collegium Medicum Jagiellonian University who met the DSM-IV criteria for major depression were enrolled. The studied population included patients with diagnosis of a first or recurrent major depressive episode. All patients demonstrated a current depressive episode. The patients received standard antidepressant therapy with venlafaxine, sertraline, escitalopram, paroxetine, fluoxetine or mirtazapine. The depressive patients were divided into two groups, that is, a treatment-sensitive (TS) and a treatment-resistant (TR) group. A TR episode was defined as a lack of remission (⩽7 points change on the 17-item Hamilton Depression scale) following a minimum of two courses of adequate antidepressant treatment (⩾4 weeks at an adequate dose). Exclusion criteria included the presence of severe and chronic medical and neurological diseases including migraine, epilepsy, Parkinson’s disease, Alzheimer’s disease, multiple sclerosis, stroke, transient ischemic attack, cerebral palsy and mental retardation, and the presence of profound personality disorders, including borderline, antisocial, narcissistic and paranoid personality disorders. The Hamilton Depression Rating Scale (HDRS, 17 items), the Clinical Global Impression scale (CGI) and the MADRS were used for assessing the efficacy of the antidepressant therapy, and patient status was evaluated before treatment and 1, 4 and 7 weeks after the initiation of treatment. Therapeutic responses were defined as ‘much improved’ or ‘very much improved’ based on the CGI scale along with a reduction of at least 50% on the MADRS (MADRS/CGI criteria) score or HADRS score (HADRS/CGI criteria). Remission was defined as ‘very much improved’ based on the CGI scale along with a score ⩽10 on the MADRS (MADRS/CGI criteria), a HADRS score (HADRS/CGI criteria) ⩽7 or a BDI score (BDI/CGI criteria) ⩽9. After the examination, venous blood was drawn. The study was approved by the Ethical Committee of Collegium Medicum Jagiellonian University Kraków, and informed consent was obtained from all participants.

### PBMC isolation

Peripheral blood was collected in K_3_EDTA collecting tubes (Sarstedt, Nümbrecht, Germany). The tubes were immediately transported to a laboratory for PBMC isolation. The lymphocytes were isolated with a Ficoll gradient (Lymphocyte Separation Medium, Lonza, Basel, Switzerland) using tubes with separation filters (BD, Franklin Lakes, NJ, USA). The PBMC layer was washed twice with PBS (Lonza), and the cells were counted and then diluted with RPMI-1640 supplemented with 10% heat-inactivated fetal bovine serum, 1% penicillin/streptomycin and 2 μm extra l-glutamine (Lonza).

### Drugs and PBMC cultures

Paroxetine (Sigma Aldrich, St. Louis, MO, USA) was dissolved in PBS at a concentration of 1 mm. PBMCs were incubated with several paroxetine concentrations (while maintaining the final PBS concentration in the medium at 4%). The cells were seeded on 96-well plates at a concentration of 2 × 10^5^ cells per well. Each drug concentration was assayed in triplicate. The PBMCs were incubated at 37 °C in 5% CO_2_ and 100% humidity and cultured for 72 h.

### WST-1 proliferation assay

The viability of the cells was examined using the WST-1 proliferation assay (Roche, Basel, Switzerland). This assay is based on the measurement of biochemical activity relative to cellular respiratory processes. A substrate (tetrazole salt) is biotransformed by mitochondrial dehydrogenase into soluble formazan and its concentrations then measured spectrophotometrically at 450 nm. Next, 5 μl of the WST-1 solution was dispensed into each cell culture well. The plates were incubated for 2 h at 37 °C. The absorbance at 450 nm was measured using a BioTek Synergy multiplate reader (BioTek, Winooski, VT, USA). The obtained results were normalized to measurements from non-treated cells and are represented as the percent growth inhibition. The drug IC_50_ values were calculated for the healthy donors using GraphPad Prism (GraphPad Software, La Jolla, CA, USA), by plotting the percent viability against the drug concentration and fitting the curve using an allosteric sigmoidal model. The PBMC-growth inhibition was calculated at the determined IC_50_ concentration using an allosteric sigmoidal curve fit. One-way analysis of variance (ANOVA) with Tukey *post hoc* and *t*-tests were used for determining the significance of differences between groups.

### RNA extraction and cDNA synthesis

Total RNA was extracted from each PBMC sample using the spin column method (RNeasy; QIAGEN, Hilden, Germany). RNA quality and quantity were checked using a NanoDrop Spectrophotometer (NanoDrop 1000, Wilmington, DE, USA) and electrophoresis in Agarose gel. One microgram of RNA was digested with gDNA Wipeout Buffer for removing genomic DNA contamination. cDNA was synthesized from RNA using the reverse transcription method (QuantiTect Reverse Transcription Kit; QIAGEN) with random oligo-dT primers.

### Quantitative real-time PCR

*CHL1.* Gene expression was determined using real-time PCR (CFX96 Touch, Bio-Rad, Hercules, CA, USA) performed with the SybrGreen I assay (Applied Biosystems, Carlsbad, CA, USA). For each reaction, 100 ng of cDNA was used. The *ACTβ* and *GUSB* genes were selected as reference genes. The temperature conditions for quantitative real-time PCR (qRT-PCR) were as follows:

- *CHL1*: 95 °C for 10 min, (95 °C for 15 s, 59.9 °C for 30 s, 72 °C for 25 s) × 55 cycles, melting analysis
- *ACTβ* and *GUSB*: 95 °C for 10 min, (95 °C for 15 s, 64 °C for 30 s) × 45 cycles, melting analysis
- The individual forward and reverse primers used were:

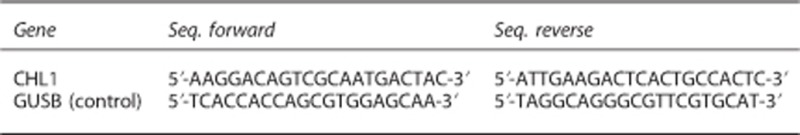


The *GUSB* gene was selected as a reference gene due to its stable expression compared with *ACTβ.*^17^ Standards and melting curves were used to control the efficiency and quality of the PCR reaction. Data analyses based on the ΔΔCt method and Bio-Rad CFX Manager software (Bio-Rad) were employed for the normalized gene study.

*ITGB3.* Real-time PCR assays were performed using TaqMan (TaqMan, Applied Biosystems) probe-based gene expression analysis for one selected gene, that is, human *ITGB3*, and one control gene, that is, human *GUSB*. The real-time PCR experiments were conducted with mixtures (20 μl final volume) containing 50 ng of cDNA template, 2 × Thermo Scientific Absolute Blue QPCR ROX Mix and 20 × Solaris quantitative PCR gene expression assays (Thermo Scientific, Carlsbad, CA, USA) with *GUSB* employed as a control gene. The PCR reactions were performed using an ABI STEP ONE Sequence Detection System (Applied Biosystems), and the cycle conditions were as follows: 50 °C for 2 min; 95 °C for 15 min; and 40 cycles of 95 °C for 15 s and 60 °C for 1 min.


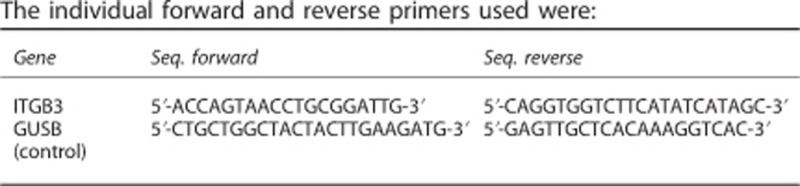


## Results

### Characteristics of the study population

The sociodemographic and clinical features of the study sample are provided in Table 1. The mean age of TS and TR MDD patients did not differ (48.4±10.9 years vs 45.1±12.3 years; *P*=0.26); no significant differences in the male/female ratios of the TS and TR MDD patients (4/29 and 7/26, respectively) were observed. While there were no significant between-group distinctions related to employment status, education and smoking, the patients with TR depression were more likely to have no partner, which agrees with earlier observations.^26^ In addition, these patients had suffered more relapses and were at a higher risk of hospitalization (Table 1).

### The MADRS scores of the patients before and following treatment

**Figure 1:**
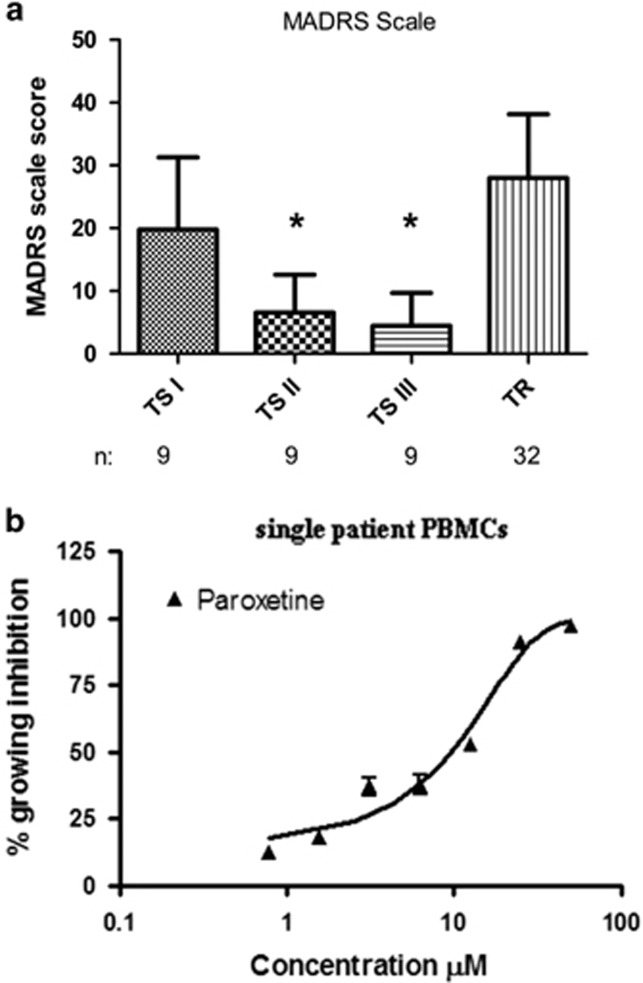
(**a**) The MADRs scores of the MDD patients in the TS I, TS II, TS III and TR groups. Only the patients who completed the entire 7-week course of treatment and donated blood three times were analyzed (significant differences were noted in the comparisons of the TS I group with the TS II and TS III groups and the comparisons of the TS II and TS III groups with the TR group; one-way ANOVA and Tukey *post hoc* tests, *P<0.05). (**b**) Paroxetine-mediated growth inhibition in PBMCs from a healthy donor (see Materials and methods for experimental protocol). The experiment was repeated three times. ANOVA, analysis of variance; MADR, Montgomery–Asberg Depression Rating Scale; MDD, major depression disorder; PBMC, peripheral blood mononuclear cell; TR, treatment resistant; TS, treatment sensitive.

The MADRS scores for the patients of the TS I, TS II, TS III and TR groups are depicted in Figure 1a, and significant improvements following 4 and 7 weeks of antidepressant drug treatment were observed. Only patients who completed the 7-week treatment and donated blood samples three times were included.

### The influence of paroxetine on PBMC viability

**Figure 2:**
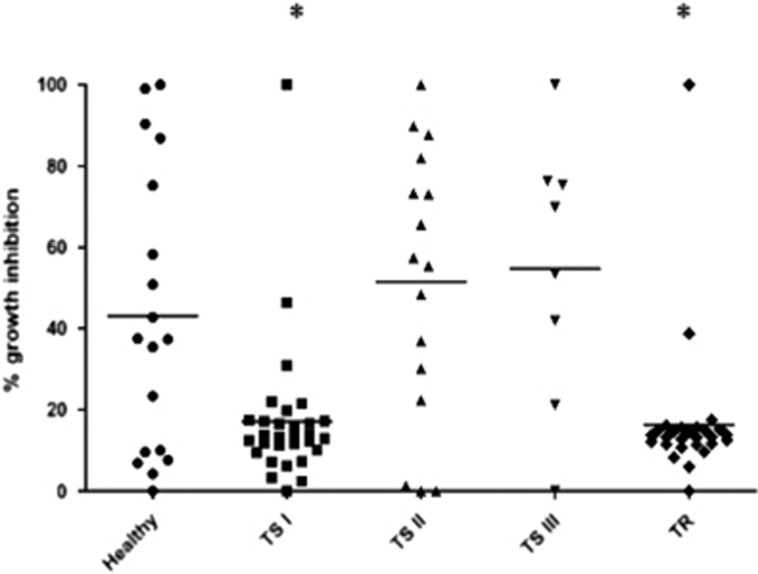
Inhibition of PBMC growth by 20 μm paroxetine in the healthy controls vs the TS I, TS II, TS III and TR MDD patients. The inhibition values for the PBMC samples from individual control and MDD patients are displayed. The lines indicate the mean values. MDD, major depression disorder; PBMC, peripheral blood mononuclear cell; TR, treatment resistant; TS, treatment sensitive.

Paroxetine inhibited PBMC growth in a dose-dependent manner with an IC_50_ of 20 μm±7.6 (Figure 1b). In the healthy control population, 20 μm paroxetine inhibited the growth of PBMCs by 46±32%. In the TS I, TS II, TS III and TR groups, the mean growth inhibition values were 13±18%, 56±33%, 62±33% and 14±16%, respectively (Figure 2). Significant differences in paroxetine growth inhibition sensitivity levels were noted between healthy and TS I groups, and healthy and TR groups (*P<*0.05; Kruskal–Wallis test and Dunn's multiple comparison test, F=23.07).

### CHL1 and ITGB3 gene expression in MDD and control PBMCs

**Figure 3:**
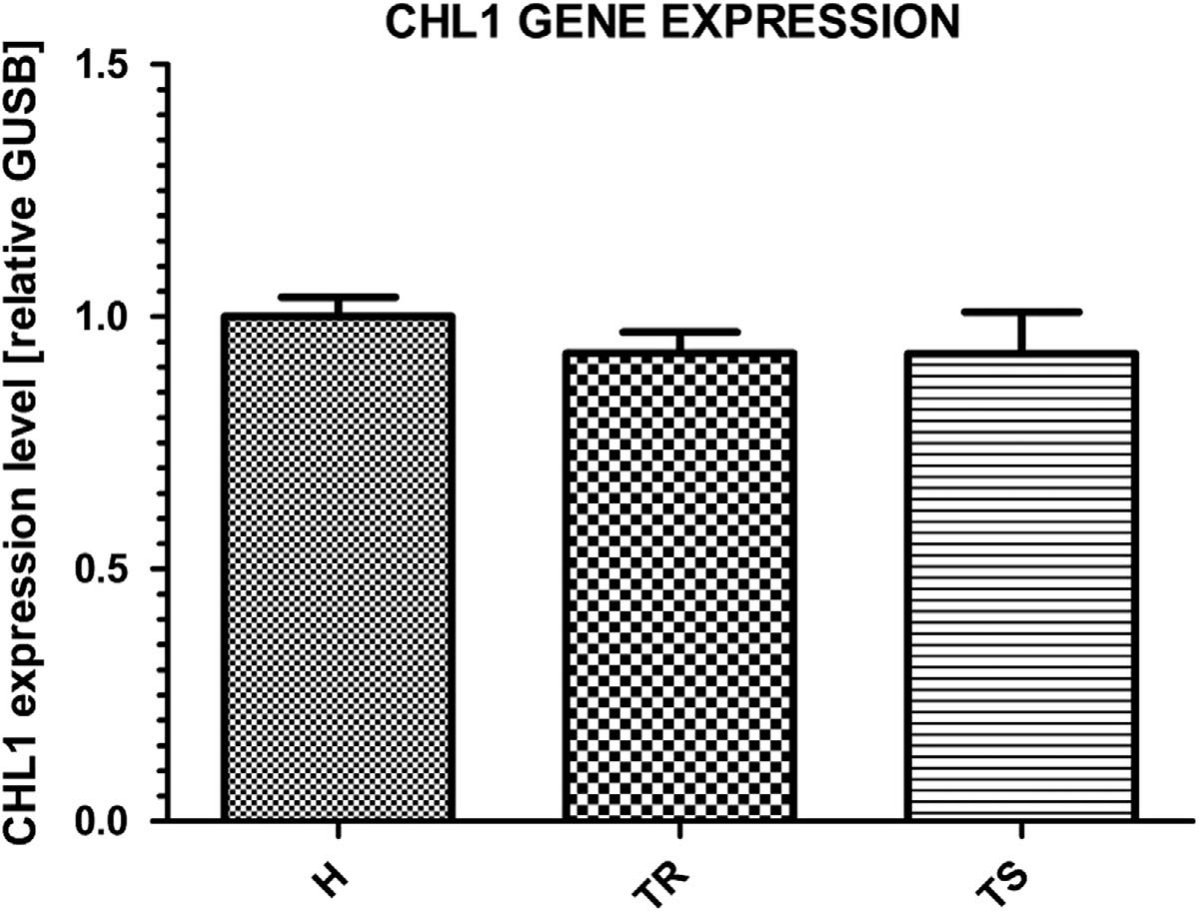
Real-time PCR measurement of *CHL1* expression in the PBMCs of the healthy controls and the two MDD groups. H, healthy volunteers; MDD, major depression disorder; PBMC, peripheral blood mononuclear cell; TR, treatment resistant; TS, treatment sensitive.

The *CHL1* gene expression level was quantified to evaluate its potential as a putative SSRI response biomarker. No significant differences in *CHL1* gene expression between investigated groups were observed (Figure 3).

**Figure 4:**
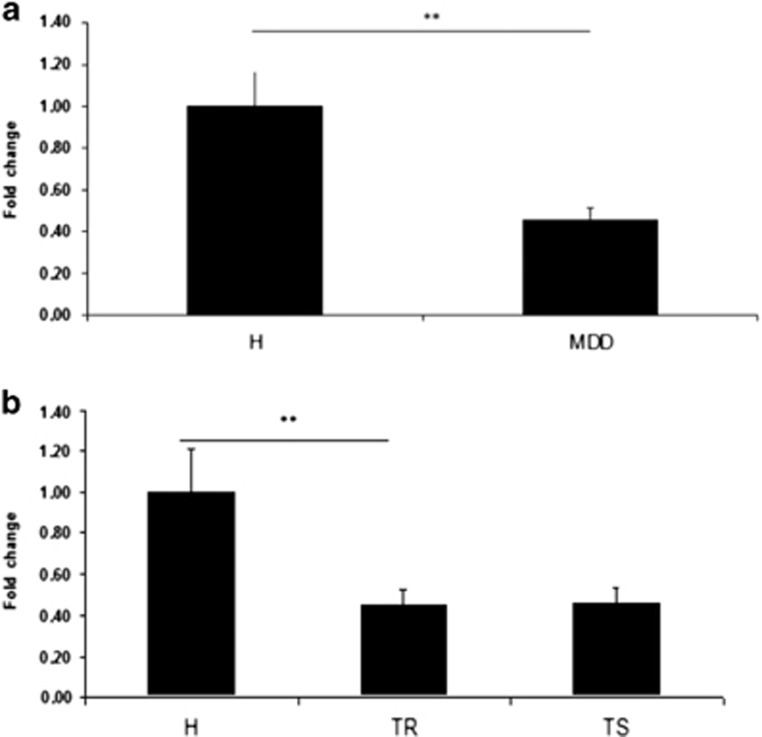
(**a** and **b**) Real-time PCR measurement of *ITGB3* expression in the PBMCs of the (**a**) healthy controls (H) and the MDD patients before treatment onset (TR+TS groups combined) and (**b**) in the healthy controls, TR and TS MDD patients. The results are expressed as fold-changes±s.e.m. for the comparisons of each group with the healthy group. **P<0.005.  *P*-values were calculated as follows: (i) Student’s *t*-test for independent samples. (ii) One-way ANOVA with Bonferroni correction. ANOVA, analysis of variance; H, healthy volunteers; MDD, major depression disorder; PBMC, peripheral blood mononuclear cell; TR, treatment resistant; TS, treatment sensitive.

We observed significantly reduced *ITGB3* expression levels in the PBMCs of the MDD patients compared with the healthy group (fold-difference: 0.45, *P*=0.0011; Figure 4a). Significantly reduced *ITGB3* expression levels were observed for the TR MDD patients compared with the control group (fold-difference: 0.45, one-way ANOVA, *P*=0.003). In addition, a trend of reduced *ITGB3* expression was observed for the TS MDD group compared with the control group (fold-difference: 0.46, one-way ANOVA, *P*=0.0712; Figure 4b).

Our findings are summarized in Supplementary Table S1. The methodology and results related to the inhibitory effects of mirtazapine are described in the Supplementary File.

## Discussion

During the past three decades, SSRI antidepressant drugs remain the first-line treatment for MDD. Yet, over 30% of MDD patients do not respond adequately to SSRI drug treatment and require alternative antidepressants, a process that may take many weeks and adds to the high morbidity and societal costs of MDD. Our study explored the potential of both phenotypic (*in vitro* SSRI sensitivity) and genomic (gene expression) measurements in PBMCs of MDD patients as potential trait and state MDD biomarkers.

Studying the influence of SSRI drugs on the growth and survival of human PBMCs is possible due to the presence of functional SERT in lymphocytes; this SERT exhibits a pharmacology that is similar to that of the SERTs in neuronal tissues and blood platelets.^9^ We observed that both the SSRI antidepressant paroxetine and the non-SSRI antidepressant mirtazapine inhibited PBMC growth in concentration-dependent manners (Figure 1b and Supplementay Figure 1S). Our findings from the PBMCs of healthy donors are closely similar to those obtained with LCLs by Morag *et al.* in ref. 14. The twofold greater paroxetine IC_50_ values of the PBMCs compared with the LCLs could reflect the fact that LCLs, unlike PBMCs, continue to proliferate *in vitro* and are therefore more sensitive to cell proliferation inhibitors. In addition, LCLs are generated from B lymphocytes, whereas PBMCs also include T-lymphocytes, monocytes and macrophages, which may also differentially affect drug-mediated growth modulation compared with LCLs.

Only a few studies by other groups have examined growth inhibition of LCLs in studies of central nervous system drugs such as lithium.^27^ The drug applied in our studies was the SSRI antidepressant paroxetine.^28^ A large spectrum of sensitivities to cell growth inhibition by paroxetine have been described in individual LCLs.^14, 15^ Our exploration of 20 μm paroxetine-mediated growth inhibition phenotypes in healthy control PBMCs revealed a large sensitivity spectrum (from full to no inhibition; Figure 2 and Supplementary Figure S3A). Among the untreated MDD patients TS I and TR, a marked significant decrease in the average sensitivity to paroxetine was observed (Figure 2 and Supplementary Figures S3B and C). In the group of 30 TS I MDD patients, only 2 patients displayed >50% growth inhibition by 20 μm paroxetine, and in the TR MDD group only a single patient exhibited similar finding. The opposite was observed in the TS II and TS III MDD groups, that is, a restoration of higher paroxetine sensitivity was observed: only 3 of 16 (19%) and 2 of 8 (25%) patients in these respective groups displayed <20% sensitivity toward 20 μm paroxetine (Supplementary Figures S4A and B). Thus, we observed that among the TS MDD patients following treatment (TS II and TS III groups), the sensitivity of the PBMCs to paroxetine-induced growth inhibition was restored in the majority of MDD patients to a level that closely resembled that of healthy control PBMCs (Figure 2), which sharply contrasts with our observations for PBMC-growth inhibition by the non-SSRI drug mirtazapine (Supplementary Figure S2).

For many years, attempts to identify trait and state biomarkers of affective disorders and for SSRI antidepressant response have met with little success. This task is difficult due to the multifactorial background of affective disorders. The perfect trait biomarker would be easily accessible, disorder-specific and constantly present (that is, independent of the symptomatic status of the patient). In contrast, an ideal state biomarker would reflect the specific phase of the disorder. Therefore, the identification of both trait and state biomarkers for affective disorders would represent significant achievement with far-reaching clinical, as well as scientific consequences, as such biomarkers would allow for better understanding of the etiological and pathophysiological correlates of affective disorders.^29^ The restoration of the lower sensitivity of the PMBCs from the TS MDD patients to the higher paroxetine-induced growth inhibition of healthy controls suggests that PBMC paroxetine sensitivity reflects a state biomarker of depression.

Both the candidate gene approach, which has, for many years, been focused on serotonin-related genes, and genome-wide association studies, which have employed SNP arrays, have been disappointing in terms of identifying antidepressant response biomarkers for personalized MDD treatment.^30^ An alternative approach, that is, genome-wide expression profiling as applied by Morag *et al.*^14^ and Oved *et al.*,^17^ has led to the identification of two brain-expressed cell adhesion protein-coding genes that are implicated in neurogenesis and synaptogenesis, that is, *CHL1* and *ITGB3.*

Expression levels of these genes in blood lymphocytes or lymphocyte-derived LCLs have been suggested as tentative biomarkers for predicting SSRI responses in MDD. Morag *et al.*^14^ reported a highly significant difference in *CHl1* gene expression between cell lines with low and high paroxetine sensitivities; however, we observed no differences in *CHL1* PBMC expression levels between the investigated groups. A recent *CHL1* genotyping study revealed associations of the rs9841789 polymorphism and its TT variant with low *CHL1* expression and high sensitivity to paroxetine.^25^ Furthermore, SNP marker analysis of a large European sample revealed a significant influence of rs2133402 alleles on drug response and remission. Treatment responses were found to be more common in patients with the G allele, and allele T was clearly more common in the non-remission patients.^25^

*ITGB3* codes for a cell adhesion protein that is of particular interest as it is essential for the activity of SERT.^23, 24^ Reduction of the efficiency of ITGB3 or the deletion of a single ITGB3 allele may result in a decrement of SERT efficacy.^24, 31^ The paroxetine-mediated inhibition of PBMC growth apparently depends on the presence of functional SERT because robust correlations of growth inhibition of LCLs from unrelated donors by different SSRIs have been observed.^13^ A significant reduction in SERT expression in the lymphocytes of MDD patients has been demonstrated,^6, 7, 11^ which may explain the PBMC group differences in paroxetine sensitivities observed in our current study. Moreover, chronic fluoxetine treatment has been demonstrated to increase the number of lymphocytes expressing SERT,^12^ which may, in part, explain the restoration of PBMC drug sensitivity observed in the majority of our TS MDD patients.

In the TR MDD patients, paroxetine inhibited PBMC growth to a lower extent. It is plausible that SSRI antidepressants are less effective in TR MDD patients because the expression and/or functionality of the SERT remains low, possibly due to the reduced expression of *ITGB3* indicated by our results (Figure 4b). The lower *ITGB3* expression observed here in MDD compared with control PBMCs is of particular interest because chronic (21 days) paroxetine treatment of LCLs has been demonstrated to increase the *ITGB3* expression by nearly twofold.^17^ Further investigation of PBMC gene expression levels following antidepressant treatment in different MDD patient groups is vital for uncovering the role of ITGB3 in the mode of action of SSRI antidepressants and the utility of PBMC *ITGB3* expression level as a MDD biomarker. The placement of our results in the context of the ITGB3/CHL1/SERT hypothesis described by Oved *et al.* in ref. 17 remains speculative and requires further investigation.

### Study limitations

The present study has several limitations. The MDD patients were treated during all stages of our study while they were taking additional antidepressant medications. Moreover, drug wash-out period was excluded due to the lack of ethical committee approval.

Future studies with larger cohorts are required to validate our current observations and should include measurements of the expression levels of additional genes and regulatory microRNAs, whose products have been implicated in the SSRI pathway, such as *SLC6A4*, miR-221 and miR-222.^17^

## Conclusion

The restoration of the sensitivity of the PMBCs from the TS MDD patients to paroxetine-induced growth inhibition may indicate that the paroxetine sensitivity of PBMCs reflects a state marker of depression. This notion is further supported by our finding that the inhibition of growth by paroxetine in the vast majority of the patients in the TR MDD group (32 out of 34) remained at the same low level observed in the untreated MDD patients. The lower *ITGB3* expression level observed in the PBMCs obtained from the MDD patients is noteworthy, and suggests that increased *ITGB3* expression on chronic SSRI treatment plays a role in the therapeutic action of these drugs in MDD.^17^ Further studies with larger patient cohorts are required to clarify the potential role of reduced PMBC SSRI sensitivity as an informative biomarker for TR MDD.

## Figures and Tables

**Table 1:** Sociodemographic and clinical characteristics of the study participants

	*TS and TR*	*TS only*	*TR only*	*Comparison TS* vs *TR (statistics)*
Mean age (±s.d.)	46.7±11.3	45.1±12.3	48.4±10.9	*P>*0.05 (*t*-test)
				
*Sex*				
Male	11(16.7%)	4 (12.1%)	7 (23.2%)	*P>*0.05 (*X*^2^-test)
Female	55 (83.3%)	29 (87.9%)	26 (78.8%)	
				
*Employment status*				
Employed	14 (21.3%)	9 (27.3%)	5 (15.1%)	*P<*0.05 (*X*^2^-test)
Unemployed	28 (42.3%)	18 (54.5%)	10 (30.3%)	
Annuitant	24 (36.4%)	6 (18.2%)	18 (54.5%)	
				
*Education*				
Primary	4 (6%)	4 (12.2%)	0	*P>*0.05 (*X*^2^-test)
Secondary	36 (54.6%)	16 (48.4%)	20 (60.6%)	
Higher	26 (39.4%)	13 (39.4)	13 (39.4%)	
				
*Marital status*				
Single	48 (72.7%)	20 (60.6%)	28 (84.8%)	*P<*0.05 (*X*^2^-test)
Married	18 (27.3%)	13 (39.4%)	5 (15.2%)	
				
*Smoking*				
Non-smokers	39 (59.1%)	19 (57.6%)	20 (60.6%)	*P>*0.05 (*X*^2^-test)
Smoking <20 cigarettes daily	13 (19.7%)	8 (24.2%)	5 (15.1%)	
Smoking >20 cigarettes daily	14 (21.2%)	6 (18.2%)	8 (24.3%)	
				
Mean number of hospitalizations (±s.d.)	2.71±3.52	1.03±2.21	4.51±3.75	*P<*0.01 (Mann–Whitney *U*-test)
Mean number of relapses (±s.d.)	1.71±1.04	1.36±0.74	2.09±1.18	*P<*0.05 (Mann–Whitney *U*-test)

Abbreviations: TR, treatment resistant; TS, treatment sensitive.

*P<*0.05 denotes statistical significance.
