# Supplementary material for: Decreased sensitivity to paroxetine-induced inhibition of peripheral blood mononuclear cell growth in depressed and antidepressant treatment-resistant patients
Source: Transl Psychiatry. 2016 May 31;6(5):e827–. doi: 10.1038/tp.2016.90 (PMC5545648; doi:10.1038/tp.2016.90)
Supplement: Supplementary Information [file tp201690x1.docx]

**Supplementary Information**

The same experimental procedure used to assess the inhibition of peripheral blood mononuclear cells (PBMCs) by paroxetine was also applied in studies of mirtazapine.

Mirtazapine (Sigma Aldrich) was dissolved in DMSO (Sigma Aldrich) to a concentration of 40 mM. PBMCs were incubated with mirtazapine at several concentrations, ensuring that the final concentration of DMSO was maintained at 1%. To achieve an appropriate number of data points to calculate the IC50 value, 9 concentrations of mirtazapine were examined (16, 23, 35, 53, 79, 119, 178, 267 and 400 μM). The cells were seeded in 96-well plates at a concentration of 0.2×10^6^ cells/well. Each drug concentration was investigated in triplicate. PBMCs were incubated at 37°C with 5% CO_2_ and 100% humidity and cultured for 72 hours.

**Results of the mirtazapine study:**

*The influence of mirtazapine on PBMC viability*

When PBMCs were incubated with different concentrations of mirtazapine, dose-dependent inhibition of cell growth with an IC_50_ of 200 μM ± 104 μM was observed (Fig. S1).

*Inhibition of human PBMC growth by mirtazapine in the control and MDD patient groups*

In the healthy control population, 200 µM mirtazapine inhibited the growth of PBMCs by 46 ± 22%; in the TS I, TS II, TS III and TR groups, the mean growth inhibition values were 46 ± 22%, 74 ± 25%, 68 ± 29%, and 34 ± 23%, respectively (Fig. S2). The changes were not statistically significant.

**Discussion:**

Mirtazapine is a [noradrenergic and specific serotonergic antidepressant](http://en.wikipedia.org/wiki/Noradrenergic_and_specific_serotonergic_antidepressant) (NaSSA), with a broad profile of receptor activities [1]. Although it has a different pharmacology compared with the SSRI drug paroxetine, mirtazapine has a similar influence on SERT activity in peripheral blood lymphocytes [2], and in our current study, it exhibited a similar inhibitory effect on the growth of PBMCs (Fig. S2A). Drug concentrations equivalent to IC_50_ values were chosen, as this approach facilitated the parallel study of several drugs in multiple patients. For mirtazapine, a large spectrum of in vitro sensitivity phenotypes was evident in the healthy controls, treatment-sensitive MDD patients prior to drug treatment (TS I), treatment-sensitive MDD patients after 4 weeks of treatment (TS II) and MDD patients after 8 weeks of treatment (TS III) as well as in treatment-resistant MDD patients (Fig. S2). The differences between the average mirtazapine-mediated growth inhibition values were not statistically significant.

*Extended view of the inhibition of human PBMC growth by paroxetine in MDD patients and controls*

In the healthy control population (22 donors), the spectrum of PBMC growth inhibition by paroxetine was observed to vary from weak or no inhibition to profound inhibition (Fig. S3A). In the group of 30 depressed patients, before treatment, paroxetine inhibited the growth of PBMCs by 100% in a single patient, whereas in 17 patients, the inhibition was 20% or less (Fig. S3B) and was almost identical to the pattern of inhibition displayed by the treatment-resistant population (Fig. S3C). After 4 weeks of treatment, the PBMC growth inhibition normalized, showing a pattern that was similar between the control group (Fig. S4A) and a group of patients treated for 7 weeks (Fig. S4B); however, this group included only 8 patients.

Our findings are summarized in supplementary Table S1.

References:

[1] de Boer T. The effects of mirtazapine on central noradrenergic and serotonergic neurotransmission. *International Clinical Psychopharmacology* 1995; **10** Suppl 4:19-23.

[2] Peña S, Baccichet E, Urbina M, Carreira I, Lima L. Effect of mirtazapine treatment on serotonin transporter in blood peripheral lymphocytes of major depression patients. *International Immunopharmacology* 2005; 5: 1069-76.

Supplementary figure and table legends:

Figure S1

Growth inhibition of PBMCs by mirtazapine. The data are representative of 3 similar experiments.

Figure S2

Inhibition of PBMC growth by 200 μM mirtazapine in healthy controls vs. TS I, TS II, TS III and TR MDD patients. Inhibition values in PBMC samples from individual control and MDD patients are displayed. Lines show the mean values.

Figure S3

Paroxetine (20 μM)-dependent growth inhibition of PBMCs from (A) healthy controls; (B) treatment-sensitive MDD patients (before treatment onset) and (C) treatment-resistant MDD patients.

Figure S4

Paroxetine (20 μM)-dependent growth inhibition of PBMCs in (A) TS II and (B) TS III MDD groups.

Table S1

Key findings and summary.
