# Supplementary figures and images for: Decreased sensitivity to paroxetine-induced inhibition of peripheral blood mononuclear cell growth in depressed and antidepressant treatment-resistant patients
Source: Transl Psychiatry. 2016 May 31;6(5):e827–. doi: 10.1038/tp.2016.90 (PMC5545648; doi:10.1038/tp.2016.90)

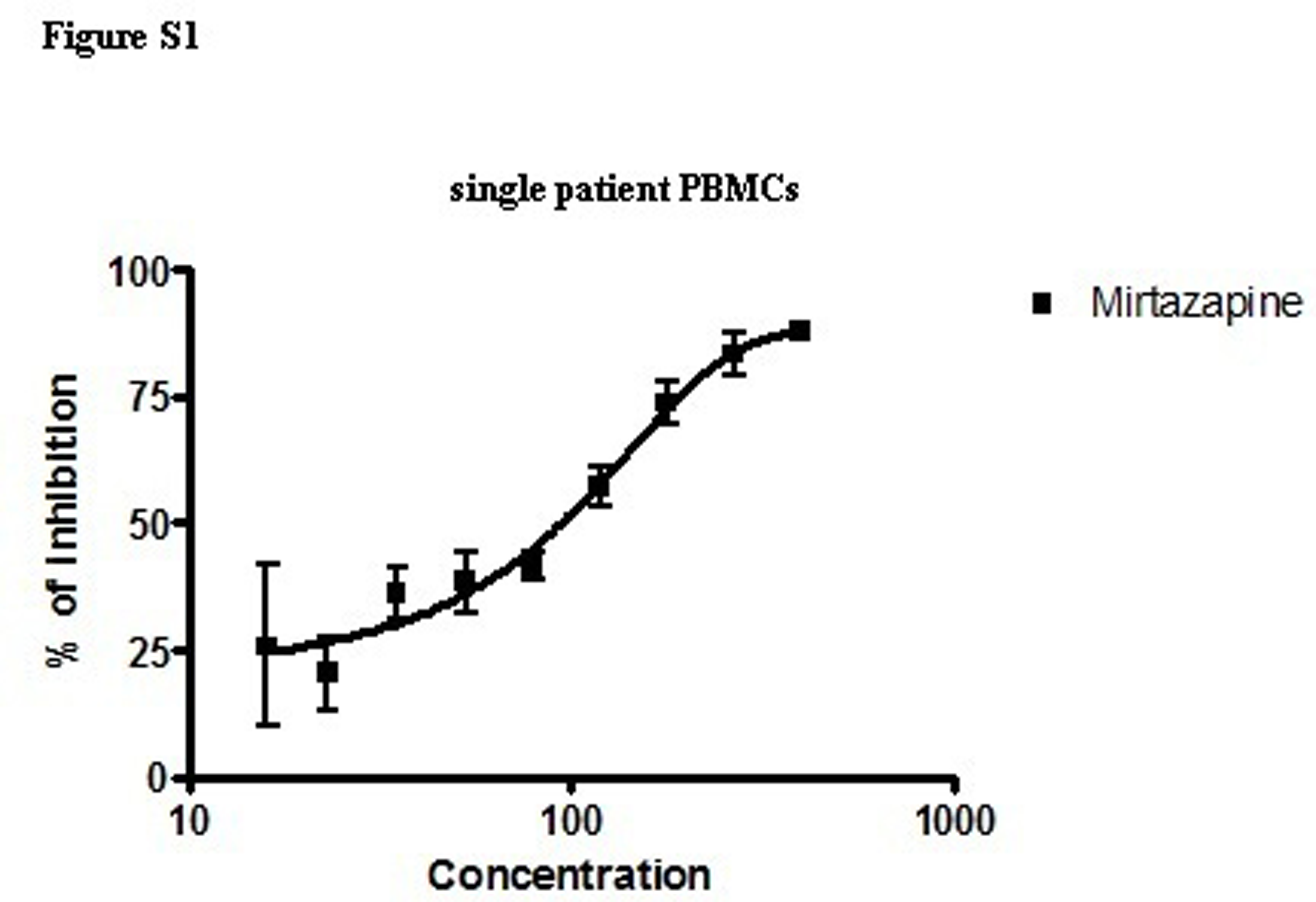

Supplement: Supplementary Figure 1 [file tp201690x2.tif]

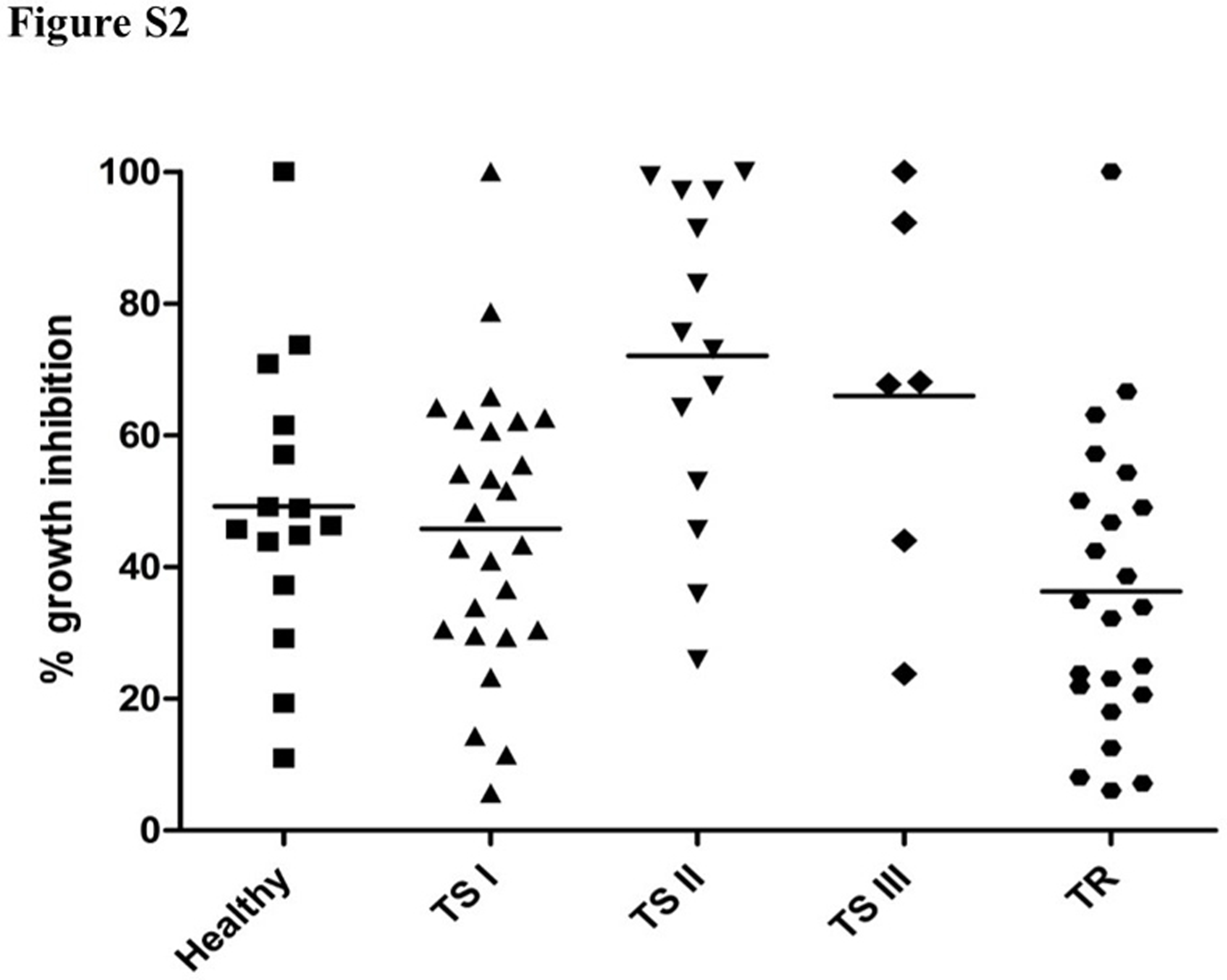

Supplement: Supplementary Figure 2 [file tp201690x3.tif]

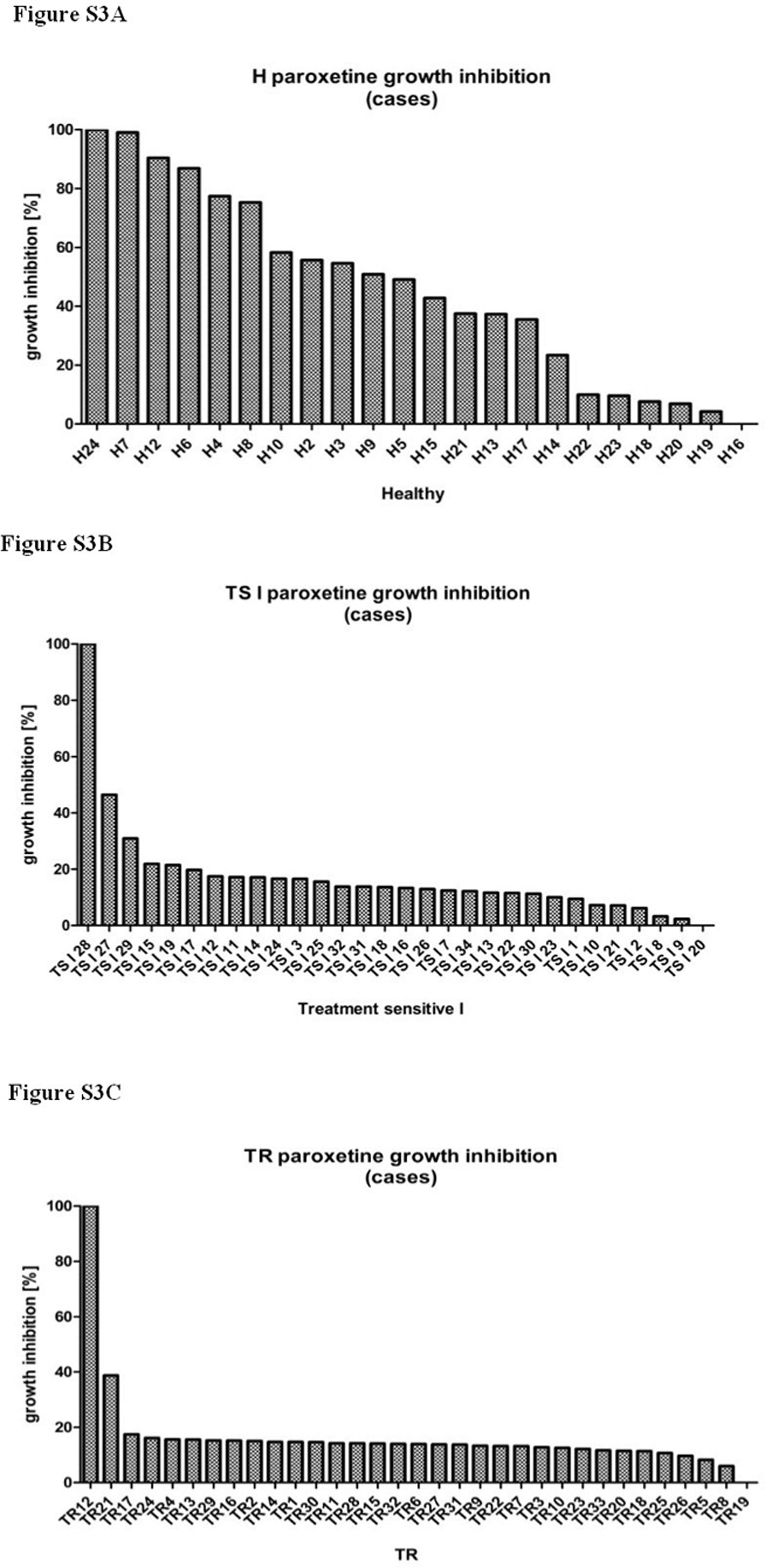

Supplement: Supplementary Figure 3 [file tp201690x4.tif]

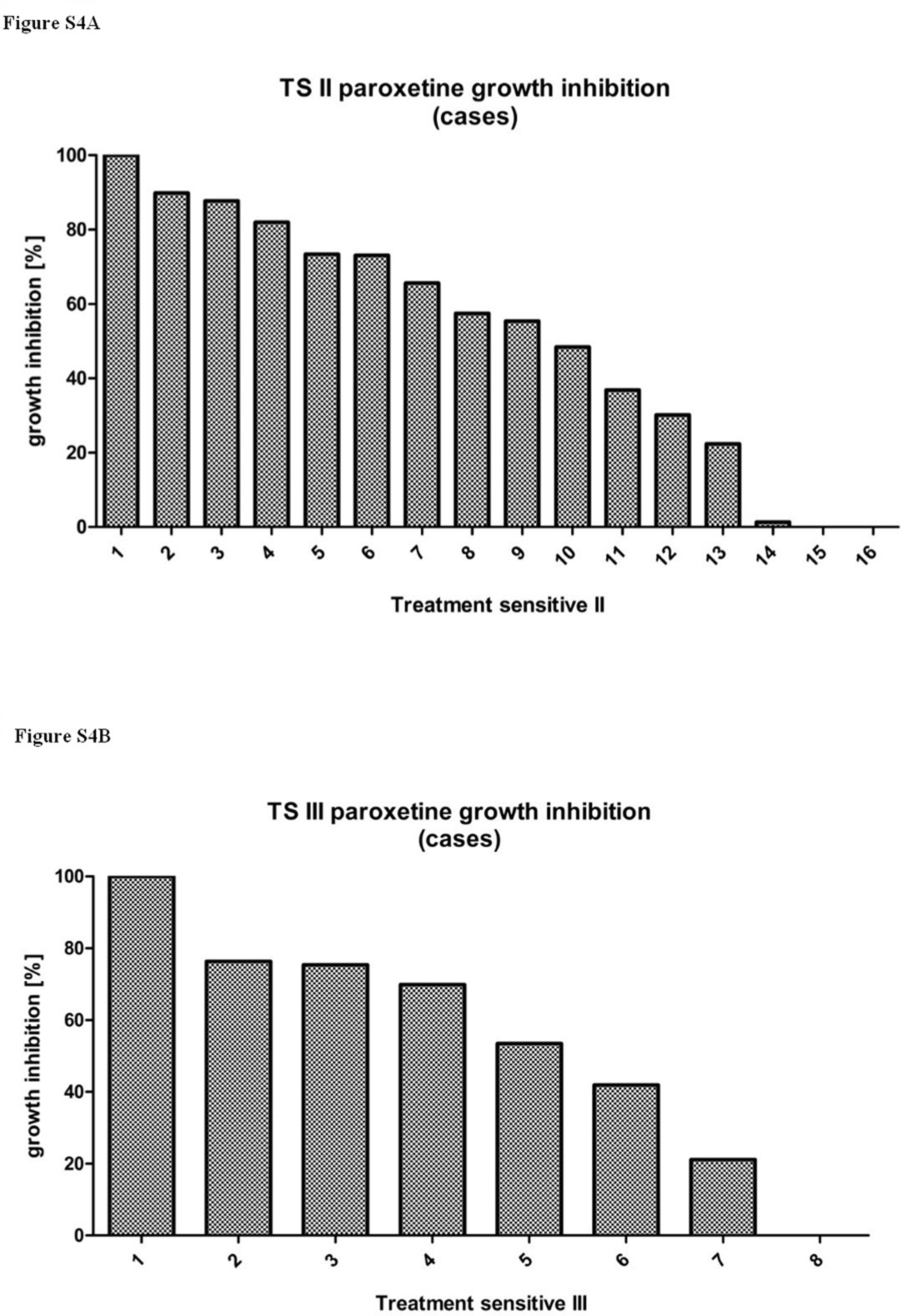

Supplement: Supplementary Figure 4 [file tp201690x5.tif]
